# Veterinary students' usage and perception of video teaching resources

**DOI:** 10.1186/1472-6920-11-1

**Published:** 2011-01-10

**Authors:** Amanda L Roshier, Neil Foster, Michael A Jones

**Affiliations:** 1 University of Nottingham, School of Veterinary Medicine and Science, Sutton Bonington, Loughborough, LE12 5RD, UK

## Abstract

**Background:**

The purpose of our study was to use a student-centred approach to develop an online video learning resource (called 'Moo Tube') at the School of Veterinary Medicine and Science, University of Nottingham, UK and also to provide guidance for other academics in the School wishing to develop a similar resource in the future.

**Methods:**

A focus group in the format of the nominal group technique was used to garner the opinions of 12 undergraduate students (3 from year-1, 4 from year-2 and 5 from year-3). Students generated lists of items in response to key questions, these responses were thematically analysed to generate key themes which were compared between the different year groups. The number of visits to 'Moo Tube' before and after an objective structured practical examination (OSPE) was also analysed to provide data on video usage.

**Results:**

Students highlighted a number of strengths of video resources which can be grouped into four overarching themes: (1) teaching enhancement, (2) accessibility, (3) technical quality and (4) video content. Of these themes, students rated teaching enhancement and accessibility most highly. Video usage was seen to significantly increase (P < 0.05) prior to an examination and significantly decrease (P < 0.05) following the examination.

**Conclusions:**

The students had a positive perception of video usage in higher education. Video usage increases prior to practical examinations. Image quality was a greater concern with year-3 students than with either year-1 or 2 students but all groups highlighted the following as important issues: i) good sound quality, ii) accessibility, including location of videos within electronic libraries, and iii) video content. Based on the findings from this study, guidelines are suggested for those developing undergraduate veterinary videos. We believe that many aspects of our list will have resonance in other areas of medicine education and higher education.

## Background

Undergraduate medical courses commonly use video technology as a source of teaching and learning and many studies have reported sound pedagogical reasons to do so. Video usage not only consolidates traditional learning resources [1] but may also provide a specific learning resource (e.g. show a practical technique) [2], while enhancing student engagement [3] and promoting deeper learning [4]. Videos may also be used for different reasons in medical training including: problem based learning [5], reviewing practical laboratory techniques [6,1,2] and observing live surgical procedures via video links [7]. Video has been used to assess veterinary students' assimilation of pre-clinical learning and communication of this knowledge in a clinical context when talking to a client [8]. Video is also a unique medium for teaching undergraduates how to take patient histories or conduct interviews [9] and how to perform clinical examinations [10]. Videoing students may also be used to heighten student awareness of their own strengths and weaknesses when obtaining clinical histories which may then increase confidence and self esteem [11,9].

Traditionally, medical teaching via videos and CD-ROMs has been delivered in audio-visual centres specific to subject areas or establishments. However, internet based platforms enable online presentation which can be accessed by students more conveniently. Increasingly online resources are being developed by the academic trainers themselves. However, if these resources are to be utilised by students a rational approach is to first find out what the students themselves require from the technology both in terms of the type of technology used and the information delivered within it.

It is worth defining the terminology we will use for evaluating videos as different terms have been used throughout the literature. The terms we will use are content, quality and clarity. By '*content' *we refer to the message the video conveys and the presentation of factual information or practical demonstration content. We use '*quality' *to refer to the technical aspects of the video such as sound, image quality, appropriate use of camera angles etc. '*Clarity' *is the term we will use to describe how well the intended message of the video is conveyed.

A number of studies have attempted to aid the production of videos in both academic institutes and the commercial sector. A study by Brown (1985) [12] highlighted the importance of video quality and targeted videos to cover learning objectives, according to the cognitive level of the students who would use them. Since this view is in accordance with constructive alignment [13] and cognitive hierarchy [14], it would appear to have a sound pedagogical basis. However, students from different year groups may obtain different learning experiences from watching the same video, according to their relative experiences (cognitive development) as knowledge becomes assimilated and accommodated within existing cognitive frameworks [15]. Therefore, rather than the structure of the video being set to a lower or higher cognitive level as described by Brown (1985) [12] the students own cognitive development may determine the learning outcomes they obtain from it. Gul *et al.*, (1999) [7] also reported that undergraduate medical students value the clarity of message rather than the quality of video presented, when viewing live video links from operating theatres. This indicates that the requirement of students may change not only according to cognitive development but also according to the type of information being delivered and in what context. In this context veterinary medicine has some unique requirements and videos have been used to highlight safe animal handling [1,2], species-specific welfare issues [16] and disease [17,6]. Other reported uses of video technology in veterinary medicine are comparable with other fields of medicine, such as use in anatomy and surgery [18,19] and pathology [20] teaching.

Another consideration when developing video technology as a student resource is its potential overuse. This is particularly important when developing easy access, online, platforms which may detract from its educational purpose. To address this problem Ellaway *et al.*, (2005) [21] have proposed four criteria, defined as the four-C's, for which learning environments should aim. These are; Convenience (Access), Consolidation, Communication and Community [21]. These criteria were developed specifically for online learning and support of the online Edinburgh veterinary curriculum which had been adapted from a similar programme used in the Edinburgh medical school. Ellaway *et al*., (2005) [21] concentrated on organisation, delivery and usage of technological platforms but did not elicit responses from those most affected by their implementation, the students themselves. However, other studies have attempted to elucidate the robustness of the four-C's concept. For example, convenience (access), with respect to the use of live video streaming of surgical procedures reported that students had a greater acceptance of technology compared to traditional teaching (observing the procedure in the operating theatre) [7]. Access is also an issue for staff, as highlighted by studies on developing online libraries of images for veterinary education [22] and it is clear that although the expansion of video technology has opened up a range of options for teaching, the management of these resources can be complex. Consolidation of learning in veterinary medicine has also been highlighted in studies linked to training in animal handling [23,2] and reported consolidation of knowledge of practical skills, either by providing pivotal information [2] or consolidation of key learning objectives [1]. The use of video to enhance sessions and delivery of material is an important feature of clinical education and other studies have shown that video can enhance learning when applied in the correct context [24,25,6]. In most studies the technologies used, and in most cases how they were used, was pre-determined without fully considering the community (stakeholders) prior to the establishment of the resource. To develop videos which have rational pedagogic perspective, it seems reasonable to first co-opt the opinion of the stakeholders themselves and as technologies advance action cycles can be used to periodically review and alter the resource, thus ensuring that the resource is technologically updated while maintaining the educational requirements of both students and academic institute.

The University of Nottingham School of Veterinary Medicine and Science (SVMS) has recognised that accessibility of videos is important and a platform to house videos was developed using space on the existing web based virtual learning environment (WebCT or Web Course Tools) [26]. This space is called 'Moo Tube', a play on the name of the popular internet video sharing resource 'You Tube' [27] and an indication that the School's video platform would share features of 'You Tube' such as a facility for rating and commenting on videos; additional features included video download facilities and quizzes specifically related to the videos. The aim of these features was to broaden the student's learning experience when accessing teaching videos. 'Moo Tube' was launched on the 20^th ^Feb 2009 and featured 22 videos made by the School. Prior to the launch of 'Moo Tube', videos were available via a list of links on the School intranet or viewed during teaching. Video development is ongoing and videos made in house were released as follows: 5 videos (2006), 13 videos (2008) and 4 videos (2009).

The main focus of the study was to qualitatively analyse the students' perception and experience of using video as a learning resource in veterinary medicine. We asked the following research questions to guide our inquiry:

i) What is the overall student view of the use of videos in the Nottingham Vet School curriculum?

ii) Do the students hold views on the quality of videos used in the teaching?

iii) Do students only use videos if driven by assessment?

iv) Do students in different academic years highlight common themes?

The study took a holistic approach, using a focus group in the format of the nominal group technique to garner opinion of different year groups. The aim of the study was to develop a student-centred resource at Nottingham Vet School but, in the broader context, to also provide a guide for the development of these resources in other academic institutions.

## Methods

A focus group format was chosen to garner student opinions as this gives students the freedom to discuss issues pertinent to them and provides an opportunity to generate themes that we may not have considered. Focus groups utilise open ended questions which promotes discussion and encourage the group to explore and clarify their views which generates more critical information and rich data [28,29]. A variety of focus group formats exist and the nominal group technique (NGT) format was chosen as this was originally developed for group decision making [30] and is recognised as a useful tool in curriculum evaluation [31]. This method produces a richness of data and reduces the researchers influence on data [32]. The NGT is a semi-quantitative/qualitative evaluative methodology which is achieved through students generating and prioritising items. The format of the NGT is to minimise issues with group dynamics and provides all members with a voice, hence the title 'nominal' [32].

The NGT format described by Chapple and Murphy (1996) [31] was followed with the addition of a fifth step. This additional step asked questions that addressed issues pertinent to developing a video resource if the students had not raised these issues during the focus group discussion. The following protocol was used for each group:

1. ***Presentation of the task***

An overview of the study and the focus group technique was explained to students.

The groups were asked three key questions, for each question steps 2-4 were carried out:

• What are the strengths of the video resources available?

• What are the weaknesses of the video resources available?

• What should be changed and how would you do it?

2. ***Silent phase***

The first question was presented and students asked to write down their responses, silently and individually.

3. ***Item generation phase***

Students took turns to provide their answer to the question and this is written on the board by the interviewer.

4. ***Discussion and clarification phase***

Students discuss the items generated in turn. This phase enables members to clarify the meaning of the items generated and negotiate the final list. Items with the same meaning are combined and duplicates eliminated. Students can agree and disagree items. The list formed here will be used for the voting phase.

5. ***Pre-determined questions***

This stage provided an opportunity for authors to ask further questions relevant to developing a video resource where this information may not have been highlighted by the students.

6. ***Voting phase***

From the list of items generated for each question, each student was asked to individually rank 5 items from each of the lists in order of most important to least important from their perspective. Ranked items were assigned values of 5 for most important to 1 least important and the number of points per item was totalled.

Each of the focus groups was facilitated by one of the authors who all followed the same protocol; this was scripted and included timing guides to provide uniformity across the different focus groups:

1. Introduction to focus group - to all groups (may help develop rapport) (10 mins)

o Thanks!

o Explain ethics - e.g. can leave at anytime, anonymous

o Explain format of the focus group

o Outline the study - interested in student perception of all video resources provided to students on the SVMS course (the resources we direct them to).

o Student feedback, both positive and negative is important - we know the resources are not perfect!

o We don't want to take up too much of their time and therefore we will be setting time limits.

o Students need to sign consent forms.

2. Key Question: 'Strengths of the video resources available' (15 mins)

Researcher: "On your own, write a list of all the '**strengths of the video resources available' **you will have 2 minutes to do this"

o Students given 2 minutes.

o Each student in turn reads their list, researcher writes the list on the flip chart and clarifies statements (e.g. summarise as a point).

Researcher: "Do you all agree with the issues raised?"

o Students can discuss, add additional points to the lists or rephrase if the group agrees.

3. Key question: 'Weaknesses of the video resources available' (15 mins)

(process as for 2.)

4. Key question: 'If you wanted to change something, how would you do it?' (15 mins)

(process as for 2.)

**5. Prompts (15 mins): the following questions specifically ask for information on video resources. This information may have been collected already**.

Usage

• How do you access video resources? (CD, online, WebCT, DVD, 'Moo Tube')

• In what context do you view videos? (e.g. playing in the background)

• When do you watch videos? (before assessment, before a practical, after a practical, for work experience preparation)

• What do you use to watch videos? (Ipod, computer etc.)

Product

• What video quality do you prefer (unedited or well edited)

• Do you use/would you like accompanying functionality (e.g. quizzes, discussion board)

• Is it important that videos can be downloaded?

• How should videos be made available? ('Moo Tube' (accessible databases), linked to lectures)

• What additional video resources would improve course delivery?

• Is there a video resource currently not provided by SVMS that you believe would improve course delivery?

**The prompts may create additional items to be added to the lists, these should be highlighted from the list originally generated by the students**.

6. Finally... (10 mins)

*Researcher: "Without discussion, please write down what you feel the top 5 aspects for each category are (1-most important, 5 least-important); you may wish to include any additional ideas you may have. Ensure question is written on the top of the post-it (good, bad, change)"*.

Total time: 1 hr 20 mins

The interviewers guided the group interactions and recorded outcomes. The interviewers input in the discussion was minimal, this approach is referred to as 'structured eavesdropping' [33]. Where disagreement occurred between the group, participants were encouraged to clarify their thoughts [28]. The focus group concluded after 1½ hours. Following the session, all participants had the opportunity to see the list of items generated by the other groups and discuss.

### Participant details and ethics

The recommended size of a focus group is up to 8 students [31]; this number is enough to generate discussion but not too big for discussions to become fragmented. The focus group consisted of undergraduate students completing their veterinary studies at the University of Nottingham. The Nottingham Vet School was established in 2006 and the veterinary programme leads to the qualification of Bachelor of Veterinary Medicine and Surgery (BVM, BVS). The curriculum consists of five years; at the time of the study the School's student body consisted of three years of students. Students from all three years of the course were invited to participate on a voluntary basis. 12 students participated in the focus group including: three year-1 (3 female), four year-2 (1 male, 3 female) and five year-3 students (1 female, 4 male). Ethical approval for this study was obtained from the ethical review committees of the School of Veterinary Medicine and Science and the School of Education, University of Nottingham and followed the 'Revised Ethical Guidelines for Educational Research (2004)' by the British Educational Research Association (BERA) [34].

The focus group was carried out 3 months after launching 'Moo Tube' and 1 month prior to end of year assessment. Students were invited to attend the study via email communication and registered their interest to participate. Sufficient numbers of students were recruited for each year to conduct a focus group for individual cohorts of students; this format was relevant as students in different years had experienced different teaching due to on-going development of the Nottingham course.

### Data analysis

**Figure 1:**
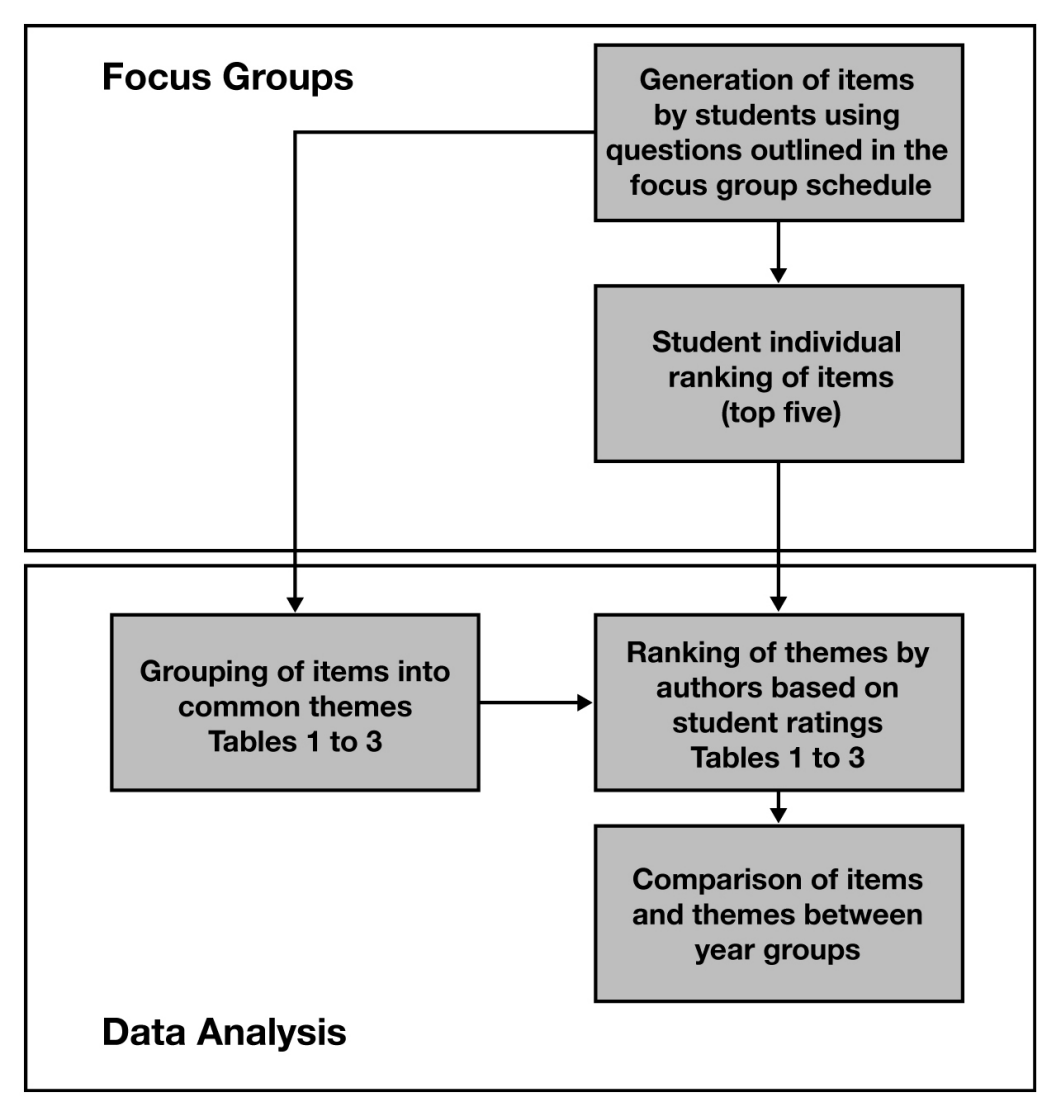
**Outline of methods**. Flowchart outlining the process of data generation, collection and analysis.

A summary of the process followed for analysing the research data is shown in Figure 1. The raw data of items identified by students were collated in a tabular format bringing the data from each year group together. A large number of items were generated and the authors thematically analysed these according to the approach of Perry and Linsley (2006) [35]. Thematic analysis required the grouping and regrouping of the items generated within the tables until no further linking by theme could be achieved. This resulted in five main themes which covered all of the items generated from each of the open questions. Establishing these themes also enabled comparison of student perception across cohorts [35]. The NGT method used also required participants to rank the top five items generated to provide an indication of a year group's most rated items. Where items had equivalent scores the number of students voting for that item was used as a deciding factor in their ranking.

### Quantitative analysis of video usage

The veterinary course is fully supported by an e-learning platform called WebCT. An analysis tool is included in the WebCT platform that enables tracking of teaching resource usage. For those videos available on the WebCT platform, an analysis of visits made to these resources was conducted and activity identified around the end of year summative assessment of practical skills. Practical skills were assessed using objective structured practical examination (OSPE) and a number of videos are directly relevant to these assessments. The number of visits to video files per day leading up to and following a practical examination were analysed to provide a quantitative measure of students accessing video resources online. Unfortunately it is not possible to measure usage of other formats of video accessed such as those that have been downloaded from 'Moo Tube' and viewed elsewhere. It is possible to analyse what downloadable video files have been accessed and these were also included in the analysis. Access of both 'Moo Tube' and videos stored on other locations of WebCT were analysed in combination.

### Statistical analysis of video usage

A χ^2 ^test (using Minitab 15 software) was performed to compare the number of times videos were accessed on each day before and after OSPE assessment (observed number) with the mean number of times videos were accessed throughout the experimental period (expected number). At P = 0.05, the tabulated value of χ^2 ^with 9 degrees of freedom was 16.92. Calculated values which exceeded this were significant (P < 0.05).

## Results

### Video usage data

**Figure 2:**
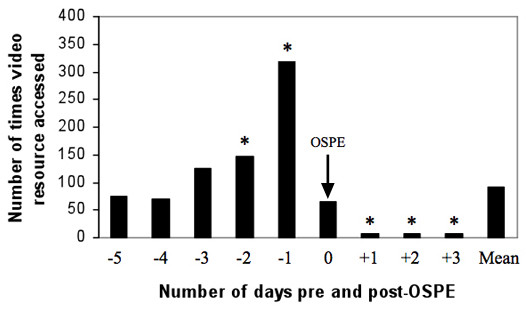
**Video usage data**. This shows the number of video accessions before and after OSPE (objective structured practical examination) (day 0). * = significant difference (P < 0.05) between the number of accessions on each day and the mean number of accessions throughout the investigation period (9 days). Arrow denotes the day on which the OSPE was taken.

Access of both 'Moo Tube' and videos stored on other locations of WebCT were analysed in combination (Figure 2). There was a significant increase (P < 0.05) in the number of times videos were accessed from 2 days prior to OSPE with greatest increase being 1 day before. On the day of the OSPE this number fell to a level which was not significant (P > 0.05) when compared with the overall mean. However, video access on each of the 3 days after the OSPE was significantly decreased (P < 0.05) when compared to the overall mean.

### Themes generated by the student focus groups

Tables 1, 2, 3 show the product of the questions explored during the focus group. The items generated by students have been grouped into themes.

**Table 1:** Student identified strengths of video use in SVMS

Theme^¥^	Year-3	Year-2	Year-1
**Accessibility**	- Videos are accessible off site and can be viewed in own time.	- Easy to find videos on the new 'Moo Tube' set up.	- 'Moo Tube'.
		- Good to go back to (in own time).	- Access.
		- A quick way to review a topic compared to reading a book.	
**Visualisation for learning**	- Presents a correct demonstration of a technique and removes staff variation in teaching.	- Clear narration, good explanations.	- Use in small group teaching room.
	- Use in lectures to visualise cases and clinical signs.	- Good to have different learning format (variety in learning). Different styles of video. Good to have for different learning style.	- Visualisation of concepts.
	- Good additional learning method.		
	- Revision and learning of skill and technique.		- Better experience/fun.

**^¥^**Themes are based on authors' categorisation of items raised by focus groups for the purpose of their grouping into common areas.

**Table 2:** Student identified weaknesses of video use in SVMS

Theme^¥^	Year-3	Year-2	Year-1
**Access**	- Lack of download opportunities.	- Inability to download video limits accessibility.	- Access to videos shown in lectures is poor.
		- Lack of indexing in long videos (i.e. like DVD chapters) - the key here is rapid accessibility to relevant material.	- Some videos are too long.
**Content**	- Some videos are 'best fit' and not filmed for purpose.	- Limited resources. Bias towards practical skills as opposed to knowledge. - Species balance - not enough large animal resources compared to small animal. - Lack of linking to other teaching resources related to the video.	- Limited number of videos available on 'Moo Tube'.
**Quality of video**	- Poor audio quality. - Poor camera technique, miss action/poor visualisation (hands cover action), angle of shot.		- Poor audio quality.
**Quality of application**	- In some cases, use of videos as a crutch to support poor teaching and are an inappropriate use of technology.		- Some videos used in lectures are not directly relevant.

**^¥^**Themes are based on authors' categorisation of items raised by focus groups for the purpose of their grouping into common areas.

**Table 3:** Student proposed improvements for video use in SVMS

Theme^¥^	Year-3	Year-2	Year-1
**Access**	- Make all videos downloadable.	- Make videos downloadable.	- Quick access to key sections in video.
	- Videos could be made in different formats and sizes, (e.g. for iPod), different streaming sizes - but in a consistent style.	- Index/summary of content within the video.	- Summary of content within the video.
		- Links to task sheets (multiple links in different places).	- Use video links in suggested reading at end of lecture.
**Content**		- More dissection/pro-section type videos - this type of video was felt to be under represented.	- Provide video of dissection.
		- Lack of linking to the teaching notes related to the video.	- More videos/practical videos.
**Quality of video content**	- Improved standards of videos produced.		
	- Training for staff - how to integrate and use videos in lectures. *(Ensure videos are appropriate tool for giving a message)*.		
	- Training for staff - how to make videos.		

**^¥^**Themes are based on authors' categorisation of items raised by focus groups for the purpose of their grouping into common areas.

## Discussion

The focus group format using the nominal group technique is a potential way of extending evaluation of students' teaching and learning experiences [31]. This format has enabled an evaluation of a teaching resource which forms the basis of the guidelines we suggest to others creating video resources. These guidelines will be discussed below.

### Strengths of video teaching in SVMS

The students from all three year groups highlighted a number of strengths which we grouped into four overarching themes (1) teaching enhancement, (2) accessibility, (3) technical quality and (4) content (Table 1). When asked to rate the items they had raised, the students focused on items from two themes; Accessibility and Enhancement. Students perceived that there was a benefit of enhancement to be gained from the use of video in learning. This correlates with the views of a number of authors who describe enhancement of learning in animal handling as benefiting consolidation of learning [23,2] and enhancing teaching [6]. The students felt that video aided in the 'visualisation of cases', provided a 'different learning method' and was useful when linked to other learning experiences. Interestingly there was a conflict in our data between the themes for strengths and weakness in that third year students recognised that some students may not like the use of video at all. Although this item was not raised by a specific member of the group, it does reflect the potential risk in using such a small group of students to identify issues. The students also highlighted items linked with good accessibility to video material in a positive light, specifically highlighting video availability through platforms such as 'Moo Tube' which offers rapid access. The students highlighted several features which indicate usage of this media at their own pace, this included downloading videos and discussion on the different times and locations for accessing videos, this indicating convenience is an important consideration for disseminating this type of resource.

It is clear from this and from other comments on the weaknesses (Table 2) and potential improvements (Table 3), that access is not perfect but that the ability to locate and access specific videos resource in their own time is important to students. Interestingly both years-1 and 2 highlighted the new 'Moo Tube' platform as a strength, while year-3 did not. A possible explanation for this finding is that 'Moo Tube' as a platform for delivery was rolled out after the year-3 students started and therefore the year-3 group may access videos via another platform. Increases in usage patterns over time was also noted by Ellaway *et al.*, (2005) [21] and this was attributed to the increase in features available and staff and students progressively engaging with the system, this would also be true of how 'Moo Tube' has developed.

### Weaknesses of video teaching in SVMS

Four key themes within the items of weakness were rated as important by students (Table 2): accessibility, quality, content and the context in which videos were used in teaching. Accessibility focused on the ability to download and links in with the idea of self-paced learning and years 1 and 2 both comment on indexing in videos to find relevant information. However, the requirement for indexing could actually indicate that a video is too lengthy. Interestingly although not in the top five issues for year-2, sound qualities were raised by all three years and was rated in the top five of both years one and three. This may relate to the importance that students have based on clarity and getting a clear demonstration for learning from the video resource.

### Areas for improvement of video teaching in SVMS

The students' rated three areas where improvements may be made; accessibility, content and quality (Table 3). All three years rate the ability to download video resources as important. However the download option is often lacking due to copyright issues on the footage. The ability to download may represent an ownership of knowledge, although there is no literature currently relating to this. The third years raised the potential of creating CD-ROMs of the School's video collection as another resource; they felt this would alleviate issues relating to video access, particularly when they were off-site. Interestingly the second years did not decide that the ability to download would supersede this requirement as they could make their own copies of CD-ROMs and allow personal cataloguing of videos. The ability to find specific videos or indexing in longer videos was raised by all three groups suggesting the tracking and locating of videos was an important issue for all students. The ability to catalogue or locate electronic resources is also an important consideration in learning as locating video resources in increasingly large databases can be a problem [22].

### Discussion of specific themes

The groups were asked about specific themes as listed in the focus group schedule. These specific questions were asked after the initial item generation process to avoid biasing student views. The year-3 group did not generate any additional comments in response to these questions as they felt they had answered them previously. Both years 1 and 2 identified that the prime usage of video material was in revision of material either for examinations or prior to practical teaching sessions relating to the video content. Evidence for this was also obtained when analysing usage trends around a practical examination (Figure 2). The students also identified additional functionality (such as quizzes and facilities to comment) as being useful and that they would like to have this option. This finding matches the responses Denwood *et al.*, (2008) [36] observed in their study on computer aided learning packages.

### Video usage pattern by students

We looked at the usage of video resources prior to practical animal handling examinations in SVMS. The data clearly shows an increase in usage in the run up to examinations and a rapid drop off after assessment, thus confirming student testaments in the focus groups that video resources were used for revision. However, the data indicated that potentially not all students were accessing video material as quite low levels of usage by the cohort were observed, although it should also be said that this type of analysis is limited by the resolution of the data we can obtain from the WebCT platform. It is not possible for us to measure video usage where an individual student downloads a specific video and multiple students view this, or students view a video as a group. In addition, it is possible that video usage scores may represent multiple access events by the same students or visits by members of staff. It was also interesting to note that students do not view all the different videos to the same extent, suggesting a pragmatic approach to their use through strategic revision. A similar variation in usage of different videos was observed by Saxena *et al.*, (2008) [19], where dependent on video, between 21 and 69% of the group would never access videos which were available to them. In our study, variation in usage may relate to the student identifying their own specific learning need but could also relate to other factors such as: the complexity of the procedure requiring greater revision, video quality (such as sound or length of video), or a lack of confidence in the method presented in the video compared to other teaching formats. Regarding decision of what methods to present on video, multiple approaches of a technique are accepted in the veterinary profession, in the focus groups students had requested a 'gold standard' approach to be demonstrated in the video. Discussions by all involved in a specific are of teaching are therefore important when creating a video resource to decide on the output as this will potentially be viewed by students as the 'gold standard'.

### Review of the research questions

We started this work with four research questions in mind. These four questions and how our data relates to these are described below.

#### (i) What is the overall student view of the use of videos in the Nottingham Vet School curriculum?

The overall view of students was positive, although weaknesses were highlighted. The positive view matches data seen in the studies of Howe *et al.*, (2005) [37] where the majority of respondents said that they felt video aided their learning and were likely to practice techniques where demonstrations were available as a video resource. Whilst respondents in the study by Gul *et al.*, (1999) [7], on telemedicine, indicated a positive preference for use of video in learning. Although our study could not assess performance linked to video usage such as that described by Rae (1993) [38] and Saxena *et al.*, (2008) [19], the respondents in our focus group indicated that video gave clarity to their learning goals for specific tasks and aided in visualisation of specific techniques.

#### (ii) Do the students hold views on the quality of videos used in the teaching?

The issue of video quality was raised in previous studies [7,10] and in these studies students regarded quality as an important feature of educational videos. However, our data suggests that video quality per se is not an important issue for all undergraduates and may be specific to the type of material being presented. In our focus groups, issues of quality were raised in both a positive and negative and there was disparity between year groups. Students from year-1 and year-2 said that the quality was good but this was not the whole picture as quality was strongly flagged up as a weakness by year-3 and more importantly all three years mentioned sound quality as an issue. The different view held by year-3 may be explained by the previously mentioned biasing in the group. However, further study would be required to ascertain if this was a major issue across the year group. This information is more useful than direct questions on specific qualities (e.g. sound, camera angle, steadiness of image, content) as we have gained insight into what students view as important issues relating to quality. The idea of quality of sound and getting a clear message from the video was flagged up by all groups, as was accuracy of content. It is possible that the provision of videos as a gold standard may over focus students on single methods which is an unrealistic representation of the real world. This point was raised by the year-3 students who may have been exposed to procedures where there is more variation in technique. In spite of the weaknesses identified, students said that some sort of video is better than nothing at all and they appreciated that creating this type of resource could present challenges for staff in terms of finding the time to create resources and training in the use of filming and editing equipment. Other studies have also found that students would tolerate some lack of quality as long as the procedure being demonstrated was clear [7]. Another factor to consider that impacts on the quality of a video is the resources available and the environment in which the video is made. Some veterinary videos are created 'in the field' where the environment may be less than optimal and where using a vast set-up of filming equipment is not practical or safe. Also, depending on the scenario being filmed, it may not be feasible to capture the perfect take such as a procedure in a barn, whereas the procedure could be explained on a model where there is time to provide explanation and set-up optimum lighting and sound. Both versions of the video have the potential to be a useful learning resource, where one captures the reality of the situation and pace of the action, the other is able to provide detail and multiple views to clearly depict the procedure.

#### (iii) Do students only use videos if driven by assessment?

The usage of videos as a revision aid was highlighted by students particularly in the use of videos for OSPE (objective structured practical examination). This trend in usage as revision aids is also observed when analysing usage data on accession of videos at key examination times (Figure 2).

#### (iv) Do students in different academic years highlight common themes?

A number of important areas for focus in the development of the video platform for SVMS were identified. We made a choice to rationalise these further into common groups. The data highlighted strengths of video at SVMS as being accessibility, and enhancement of learning. This provides evidence that the application of video resources within the School is in line with the criteria laid out by Ellaway *et al.*, (2005) [21] and Brown (1985) [12]. However students thought there was still room for improvement in the area of access as this was highlighted along with limitations in content, technical quality of videos and their application by teaching practitioners. This highlights one of the issues of the use of technology mentioned by Ellaway *et al.*, (2005) [21], that technology should not be an end in its self and may not lead to good practice. The issues of quality were highlighted by third year students and are counter to those of the other two years. This suggests that either there was a specific problem with video quality in the videos accessed by year-3 students, or that this group was more aware of issues of quality. Members of the year-3 group were experienced in developing their own video material which may impact on their expectations. All three years commented on inappropriate use of videos in teaching although it was only ranked highly by years one and three. This result aligns with the views of Ellaway *et al.*, (2005) [21] on the appropriate use of technology. Whilst this may not reflect on the quality of videos or the platforms used, it does suggest that some staff discussion or training on the matter could improve the student learning experience. The evolving nature of the new course at SVMS means that changes in the creation and presentation of video material is already occurring and may explain the different views held by students in different year groups. Student feedback is instrumental for assisting with the development of teaching videos and this feedback should be an ongoing process, particularly where curriculums are under development. All years discussed limitations in the range of video topics available such as breadth of species and types of material and highlighted this as an area for development.

It could be argued that one of the main issues with this data is the small group size from which it is collected especially within year groups sizes were smaller than the optimal group size suggested by (Chapple and Murphy 1996) [31]. They recommended groups size for focus groups of up to eight students and our year groups were three, four and five students. The small group size meant that the relative weighting to different issues by students may be skewed although there does appear to be a trend in scoring for the different issues rated top of the list for each group. However, the groupings of the data combined across year groups represents a larger number and may be more robust. We observed differences between the years, however these views could be biased by the small group size which may not reflect the cohorts opinions. Selection of students was on a voluntary basis and we know that a number of students in the year-3 group have interests in the use of video and were skilled in editing and this may be reflected in this year, highlighting production and quality issues compared to others.

The benefit of the methodology chosen to study the role of video in SVMS has been the unsolicited identification of themes. The unprompted comprehension by the focus groups of the breadth of usage of video in teaching, as outlined in our review of the literature, would suggest that if nothing else there is a general perception by students that the use of videos in teaching is good but that the use of video at SVMS and other educational establishments needs to be reviewed to ensure that it provides a useful tool for student learning. A survey of students found that technologies were used for socialising and entertainment but there was no expectation for these to be used in the learning environment [39]. However, this study was conducted in 2001 and the integration of technology in daily lives and education has developed therefore these findings may not transfer to current day. In contrast to this view, Prensky (2001) [40] describes those students who are familiar and confident users of computers and wider technologies as 'digital natives', and those older students and staff who are less familiar as 'digital immigrants'. Prensky believes that the 'digital immigrants' will need to change their ways to engage the 'digital natives'. The Nottingham Vet School is an establishment highly immersed in educational technologies, including the provision of laptops to all students under the premise that the course is provided paper free and educational resources are distributed using a virtual learning environment. Not all our students are 'technology savvy' or indeed are 'digital natives' but are supported to enable access to resources. We propose another name for students who find themselves in a technology rich environment, these are the 'technology submerged'. It is therefore imperative that educators understand the students' perspective in the use of technologies so that they are able to develop appropriate resources and provide support for each new generation of students.

### Using technology in higher education and influence on teaching practice

The successful integration of teaching technologies in higher education is dependent on the resources available for its implementation, the skill set of the operators and its necessity to the users. Barriers to integration were identified by Ertmer (1999) [41] and were referred to as first-order obstacles (e.g. equipment, training, technical support) and second-order barriers (e.g. teachers' own beliefs). From the study findings and through our experience of delivering video resources we have proposed guidelines others may wish to consider when developing video resources. We propose these guidelines with reference to Ellaway *et al.*, (2005) [21] who suggested the four C's of engagement (convenience and accessibility, consolidation, communication and community) should be considered when evaluating how technology enhances the student learning environment. In addition, we also suggest guidance for a fifth C: 'creating videos'.

### Guidelines for creating video resources

#### i) Convenience (accessibility)

1. Ensure students receive adequate training and information on educational technologies. Do not assume that all students will be familiar with technology and therefore feel comfortable accessing resources.

2. Ensure all students are provided with the information to access the video resource.

3. Consider accessibility to the resource (e.g. consider the inclusion of subtitles).

4. The ability to locate and access specific videos resource in their own time is important to students - consider how videos will be stored, accessed and catalogued.

5. The facility to download videos assists accessibility to resources. Students are then able to catalogue resources as they wish.

6. Reference to other teaching material, e.g. relevant lectures and practicals.

#### ii) Consolidation

7. Question the relevance of a video. Will it enhance the teaching or facilitate access to the information by students with different learning styles.

8. Apply knowledge of pedagogical understanding to enhance message given in video.

9. The message and content of the video is the primary consideration. Aspects of video quality can be overlooked as long as it is not detrimental to the clarity of the message.

10. Students have a preference for accessing videos prior to assessment and practical teaching sessions.

11. Students are more likely to practice practical techniques when a teaching video exists.

12. Students find that videos aid the clarification of learning goals and aids visualisation of the technique.

#### iii) Communication

13. Communication around videos can take place through many channels such as face-to-face, notice boards, Email or virtual environment facilities (chat rooms, discussion forums).

14. Consider using communication tools available in virtual environments, to encourage a more dynamic approach to viewing video. For example, the opportunity to rate videos and write comments encourages students to engage more deeply with the video material.

15. Communicate to staff and students video resource availability.

a) This will raise awareness and may aid integration of the resource in other areas of teaching.

b) Staff awareness of others developing this type of resource can then support each other, thus overcoming a potential barrier to adopting this type of resource.

c) Send alerts when new video material is available.

#### iv) Community

16. Create discussion around existing videos via:

a) discussion forums

b) instigating or encouraging a shared opportunity to watch videos

17. Encourage feedback from students and staff on videos that have been created.

18. Involve students and staff in the process of creating videos.

19. Open Access resource initiatives - share your video efforts with others within and outside your institution.

#### v) Creating videos

20. Generate basic guidelines for authors to follow when creating videos. Establish a video production process and provide guidelines to video authors. Create basic principles to follow such as guidance on video length and quality expectations.

21. Identify a key person to take ownership of the video title who will be responsible throughout the video production process.

22. Investigate any copyright or rights waiver issues/permissions/health and safety guidelines before filming.

23. Keep records of video documentation such as copyright permission and contributions for each video. This information will ensure appropriate distribution and sharing of the video resource.

24. Identify opportunities in teaching that would benefit from a video resource. Collect student opinions, what they like, what they don't like about existing video resources.

25. Take care when creating videos of gold standard techniques. Students may be frustrated if alternative approaches are also taught. If it is not possible to gain consensus on a technique, clarify why this is the case to the students.

26. Ensure the message you are trying to convey in the video is clear.

27. Plan before creating a video, check resources, create storyboards, discuss with others involved in teaching the topic of the video.

28. Avoid lengthy videos. We suggest videos length of up to 5 minutes and avoid going over 8 minutes.

29. If the video is lengthy, consider:

a) breaking the video into separate shorter videos

b) including searchable chapters

c) avoiding the use of repetition and slow motion demonstration of techniques. The video format enables students to review the footage at their own pace (e.g. to pause and rewind etc.).

30. Establish an evaluation process for all videos created or adopted. Staff and students should be involved in this process.

31. Review video resources in terms of visual and audio quality.

32. Create additional resources around a video to maximise its use and educational impact (e.g. quizzes, discussion, identify related videos).

33. Consider when to show the video. In our study, students said watching videos that are available elsewhere during lectures was frustrating. Consider part i) of these guidelines: convenience (accessibility).

34. Be aware of potential barriers for integrating videos: i) resource availability (e.g. staff with appropriate skill set to make a video, filming equipment, video editing facilities), ii) the skill set of the operators to work with video technology, and iii) the necessity of the video to the users.

35. Once you start creating videos, it is possible that the students will want you to make more and that you may raise their expectation of the resources provided by you and your colleagues!

## Conclusion

The use of the nominal group technique is a valuable tool enabling product evaluation. The students had a positive perception of video usage in higher education. Video usage increases prior to practical examinations. Image quality was a greater concern with year-3 students than with either year 1 or 2 students but all groups highlighted the following as important issues: i) good sound quality, ii) accessibility, including location of videos within electronic libraries, and iii) video content. Based on the findings from this study and through their experience of developing teaching videos, the authors have suggested guidelines for developing undergraduate veterinary videos. We believe that many aspects of our list will have resonance in other areas of medicine education and higher education.

## Competing interests

The authors declare that they have no competing interests.

## Authors' contributions

ALR conceived of the study and was responsible for co-ordinating the project and development of the manuscript. MAJ made substantial contributions to the analysis and interpretation of data. NF performed statistical analysis of the data. All authors have been involved in drafting and revising the manuscript critically for important intellectual content. All authors have made contributions to the study design, acquisition and interpretation of data and have read and approved the final manuscript.

## Authors' information

ALR: BSc(Hons), PhD, PGCHE, PgCert, MA, PgDip, FHEA Is a university teaching fellow lecturing in biomechanics and animal behaviour, and is involved in education research with a particular interest in implementing and researching education technologies.

NF: BSc(Hons), PhD, PGCHE, MA, FHEA Is a lecturer in immunology and infection and is interested in module development.

MAJ: BSc (Hons), MSc, PhD, PGCHE, MA, FHEA Is a lecturer in microbiology with interests in vaccine development, enteric disease in poultry and is involved in education research with an integrated teaching interest, assessment and feedback.

## Pre-publication history

The pre-publication history for this paper can be accessed here:

http://www.biomedcentral.com/1472-6920/11/1/prepub
